# Modeling the Impact of Proactive Community Case Management on Reducing Confirmed Malaria Cases in Sub-Saharan African Countries

**DOI:** 10.4269/ajtmh.23-0844

**Published:** 2024-07-09

**Authors:** Yifan Wang, Xingjian Wang, Brian Gurbaxani, Julie R. Gutman, Pinar Keskinocak, Hannah K. Smalley, Julie Thwing

**Affiliations:** ^1^ H. Milton Stewart School of Industrial and Systems Engineering, Georgia Institute of Technology, Atlanta, Georgia;; ^2^ National Center for Immunization and Respiratory Diseases, Influenza Division, Epidemiology and Prevention Branch, Centers for Disease Control and Prevention, Atlanta, Georgia;; ^3^ Division of Parasitic Diseases and Malaria, Global Health Center, U.S. Centers for Disease Control and Prevention, Atlanta, Georgia

## Abstract

Malaria continues to be a major source of morbidity and mortality in sub-Saharan Africa. Timely, accurate, and effective case management is critical to malaria control. Proactive community case management (ProCCM) is a new strategy in which a community health worker “sweeps” a village, visiting households at defined intervals to proactively provide diagnostic testing and treatment if indicated. Pilot experiments have shown the potential of ProCCM for controlling malaria transmission; identifying the best strategy for administering ProCCM in terms of interval timings and number of sweeps could lead to further reductions in malaria infections. We developed an agent-based simulation to model malaria transmission and the impact of various ProCCM strategies. The model was validated using symptomatic prevalence data from a ProCCM pilot study in Senegal. Various ProCCM strategies were tested to evaluate the potential for reducing parasitologically confirmed symptomatic malaria cases in the Senegal setting. We found that weekly ProCCM sweeps during a 21-week transmission season could reduce cases by 36.3% per year compared with no sweeps. Alternatively, two initial fortnightly sweeps, seven weekly sweeps, and finally four fortnightly sweeps (13 sweeps total) could reduce confirmed malaria cases by 30.5% per year while reducing the number of diagnostic tests and corresponding costs by about 33%. Under a highly seasonal transmission setting, starting the sweeps early with longer duration and higher frequency would increase the impact of ProCCM, though with diminishing returns. The model is flexible and allows decision-makers to evaluate implementation strategies incorporating sweep frequency, time of year, and available budget.

## INTRODUCTION

Globally, there were an estimated 247 million cases and 619,000 deaths due to malaria in 2021, primarily among children under 5 years of age in sub-Saharan Africa.^1^^,^^2^ After a decades-long downward trend, the burden of malaria morbidity and mortality has plateaued in the last few years; there were an estimated 212 million malaria cases in 2015.^3^ Interventions for malaria prevention and control include insecticide-treated nets, indoor residual spraying, intermittent preventive treatment of pregnant women, seasonal malaria chemoprevention, and malaria case management (i.e., parasitologic diagnosis by malaria microscopy or rapid diagnostic test (RDT) and treatment of cases with artemisinin-based combination therapy [ACT]).^4^ Timely, accurate, and effective case management, particularly diagnostic confirmation of suspected cases and appropriate treatment within 24 hours of symptom onset, is critical to malaria control. Early detection and treatment reduces the severity of symptoms and may reduce the parasite reservoir in a community.^5^ However, barriers such as geographic distance to healthcare facilities, availability of affordable transportation, costs in terms of both financial resources and lost wages, and perception of risk hinder care seeking and thus coverage of malaria case management.^6^ Even as recently as 2015, fewer than 20% of children with malaria in endemic zones were reported to have been treated with an ACT.^7^

Malaria community case management (mCCM), known in Senegal as *prise encharge a domicile*, or PECADOM, was introduced in Senegal to address barriers to receiving prompt care.^8^ In this model of malaria case management delivery, villages at least 5 km from the nearest health facility were selected, and a resident of the village was selected as a community health worker (CHW) and trained to diagnose malaria with RDT and treat those with positive tests with an ACT. Residents were thus able to seek care with the CHW for malaria diagnosis and treatment within the community. An evaluation based on routine surveillance data collected by the CHWs and at health facilities showed that intervention regions experienced a statistically significant decrease in all deaths and deaths attributed to malaria, whereas no such decrease was seen in comparison regions.^9^

Despite the scale-up of mCCM, poor care seeking and limited access to care at the community level continued to be observed, prompting the development of a proactive model. In addition, CHWs had challenges with frequent stockouts of RDTs and ACTs^10^ and resultant lack of community confidence.^11^

Proactive community case management (ProCCM, or PECADOM Plus in Senegal) is a novel strategy for delivering malaria case management, in which a CHW visits every household in the community (“sweeps”) regularly and frequently to identify individuals who display malaria symptoms and administers an RDT to those with symptoms of malaria and an ACT to those who test positive. Patients who display danger signs, such as impairment of consciousness, prostration, or inability to eat or drink, are urgently referred to a nearby health facility. The potential advantages of ProCCM are early intervention for better health outcomes in malaria cases and the potential to reduce the proportion of the population with gametocytes, which in turn has the potential to reduce future incidence of malaria and the overall proportion of the population infected.

**Figure 1.**
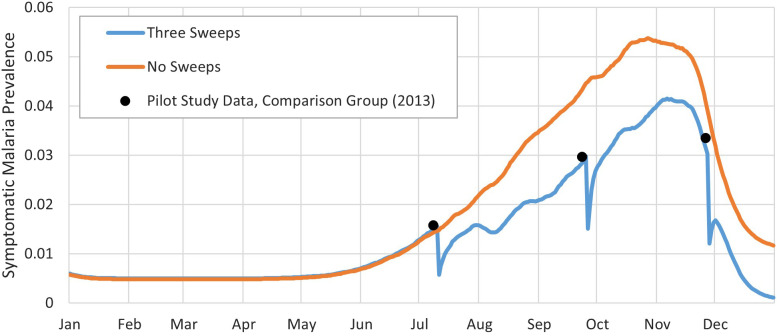
Symptomatic malaria prevalence simulation results for Strategy B from Table 1 (three sweeps, at baseline, midline, and endline of high-transmission season).

**Figure 2.**
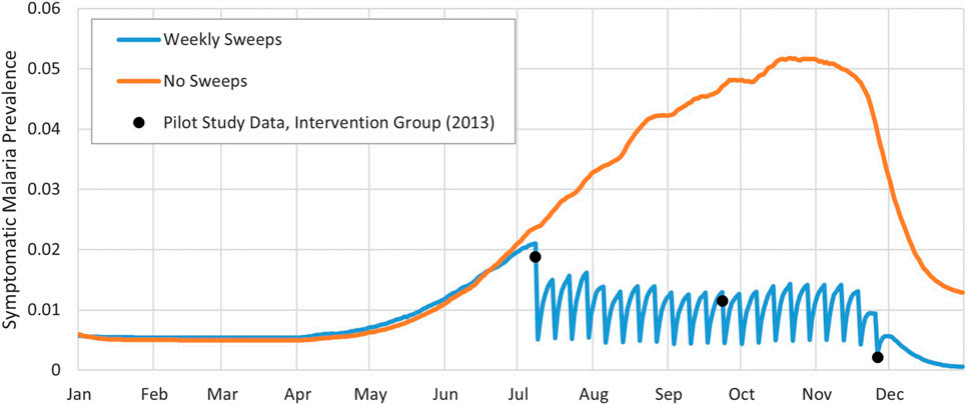
Symptomatic malaria prevalence simulation results for Strategy C from Table 1 (weekly sweeps × 21 weeks).

Proactive community case management was piloted in the Saraya district in the Kédougou region of Senegal, which has one of the highest childhood mortality rates (74 deaths per 1,000 live births) and the highest prevalence of *Plasmodium falciparum* (15.3%) among children under 5 years of age in Senegal.^12^ Community-case management of malaria (PECADOM) was introduced in 2008 in 20 villages in Saraya and expanded to more than 800 villages in 2010.^13^ However, although community care providers were trained and available to community members under this model, they did not visit households, and care seeking remained suboptimal, resulting in preventable morbidity and mortality. Thus, PECADOM was revised to include household visits by CHWs. Proactive community case management, also known as PECADOM Plus, was piloted in one village in 2012 and then studied in 15 intervention villages and 15 comparison villages in a 2013 trial in Kédougou, Senegal.^11^ Although CHWs were present in all study villages, in the comparison group, only three home visits were conducted during the first weeks in July, September, and November, whereas in the intervention group, weekly sweeps were conducted by CHWs during the entire malaria peak season (from July to November, for 21 weeks). At the end of the trial, the proportion of the population with symptomatic, confirmed malaria infection was 16 times higher in the comparison villages (those that received household visits only at the beginning, middle, and end of transmission season) than in the intervention villages (which received weekly household visits). Adjusting for potential confounders, the ProCCM intervention was associated with a 30‐fold reduction in odds of symptomatic malaria in the intervention villages at the end of the trial (adjusted odds ratio = 0.033; 95% CI: 0.017, 0.065; see also Figures 1 and 2 for modeled performance of the intervention and comparison strategies). The total reduction in malaria incidence over the course of the trial was more modest (estimated reduction from approximately 3,700 to 2,350 cases). The ProCCM strategy was adopted by the Senegal National Malaria Control Program after the trial. Scale-up started in 2014 with 132 villages in the Kédougou region, and as of 2018, included 1,944 villages in 10 regions.^13^

A number of studies examining the potential impact of ProCCM compared with conventional case management are underway or have recently been completed. Although ProCCM delivers promising benefits in general, different implementation strategies in terms of sweep timing and frequency (depending on limited resources) led to differences in ProCCM effectiveness.^11^^,^^14^^,^^15^ In Senegal, scale-up was rapid after the initial pilot study, and data collected for programmatic monitoring have lacked sufficient granularity to track impact. This paper aimed to find the best strategy for administering ProCCM in south Senegal with the goal of developing insights that could be generalized to other similar environments. By developing a methodology to predict the impact of various ProCCM strategies, we were able to identify essential factors influencing the effectiveness of ProCCM and construct an optimized strategy considering the resources required. We built a simulation that models the mosquito population, the transmission from mosquitoes to humans, and transitions between different stages of infection in humans based on published frameworks,^16^^–^^18^ and we validated the model using the data from the pilot.^11^ We used the model to test various strategies for implementing ProCCM in Senegal (e.g., with different combinations of coverage and visit frequency) and compare outcomes such as malaria incidence and cases averted as a result of the intervention.

## MATERIALS AND METHODS

We used an agent-based simulation model that incorporated the dynamics of the mosquito population over time using differential equations, the progression of malaria within humans with simulated humans tracked individually (i.e., as individual agents with attributes that characterize their current state), and transmission dynamics between humans and mosquitos to evaluate ProCCM strategies. The study followed the Standards for Quality Improvement Reporting Excellence (SQUIRE) guidelines.^19^

### Data.

The majority of the data for the simulation model (Supplemental Appendix, Section 1, Supplemental Table 1) comes from the Saraya Health District, with a total population of 52,590 full-time residents.^20^ Proactive community case management was piloted under two scenarios: weekly sweeps (intervention group) and three sweeps (comparison group; see Supplemental Appendix, Section 1, Supplemental Table 1 for details).^11^ The data collected during the ProCCM pilot was restricted to the population of the villages, number of fevers (from any cause) detected by CHWs, number of RDTs performed, and number of positive RDTs per village weekly during the sweeps.^11^^,^^20^

### Agent-based simulation model.

We developed a hybrid model that simulates the dynamics of the mosquito population with ordinary differential equations using the general framework proposed in Cailly et al.^16^ and Tran et al.^17^ and the dynamics of humans with more complex agent-based models based on Winskill et al.^18^ Model details are included in Supplemental Appendix, Section 2. In the simulation, if a symptomatic human seeks treatment at any stage after developing symptoms, we assume that he/she will receive ACTs^10^ and that the recovery process starts immediately after the first dose of antimalarial drug.^21^ Treatment outcome depends on how long one had been infected as well as the severity of symptoms (Supplemental File, Section 2.4).^22^ Traditionally, an infected human would only actively seek treatment if he/she developed symptoms. Those who do not seek treatment will keep infecting or even superinfecting mosquitoes. Proactive community case management could actively detect infected humans with symptoms, give them treatments, and reduce the parasitic load in the ecosystem. Because we modeled the spread of malaria in a region where malaria transmission is seasonal and the modeled transmission of malaria could theoretically die out, the model reintroduces malaria each new season. Handling chronic asymptomatic and minimally symptomatic carriers in such a way will not impact the number of symptomatic cases because, during the dry season, chronic asymptomatic individuals will not spread malaria owing to the nonexistence of mosquitoes.^23^

### Model validation.

We validated our model as much as our dataset would allow by comparing the model results with the data collected during the ProCCM pilot.^11^ Outcomes were calculated in terms of RDTs performed, proportion of the population with symptomatic confirmed infections (positive RDTs/population) per sweep, and cases averted. The simulation results, including the baseline case of no sweeps, are shown in Figures 1 and 2, representing three sweeps and weekly sweeps, respectively.

Based on the mean of 50 replications, our model showed consistency with the malaria infection data from the pilot study; the data points from the pilot study lie within the 95% CIs of our simulation results (Supplemental Appendix, Section 3).

### Simulation model runs.

After validation, we ran our simulation model considering ProCCM implementation strategies used during the pilot study (i.e., no sweeps, three sweeps, or weekly sweeps with 100% coverage during the 21 weeks of the malaria transmission season [ProCCM strategies A, B, and C, respectively, in Table 1]). We also considered variations to these strategies with respect to number of sweeps, delay between sweeps, coverage (i.e., percentage of households visited during a sweep), and start/end dates of sweep strategy (Table 1). For each strategy, we performed 50 replications and recorded the mean number of year-round symptomatic malaria infection cases, peak percentage of humans with symptomatic malaria infections (peak symptomatic malaria infection prevalence) during a given sweep, the total number of RDT tests used during ProCCM sweeps, and the corresponding SDs of the means. Each simulation run spanned 2 years, including a warm-up period of 1 year. Note that both rolling mean and SD of symptomatic cases stabilized after approximately 40 replications. To check model results, we also conducted a sensitivity analysis of the ProCCM sweep coverage and simulation year. We compared the number of year-round symptomatic (usually febrile illness) malaria infection cases and peak proportion of the population with symptomatic malaria infection detected during the sweep under the different sweep strategies. For additional details, see the Supplemental Appendix, Section 3.

**Table 1:** List of all simulated ProCCM strategies

ProCCM Strategy	Coverage	No. of Sweeps	Sweep Period Duration
A. No Sweeps	–	0	–
Implemented Strategies			
B. Three Sweeps	100%	3	21 Weeks
C. Weekly Sweeps	100%	21	21 Weeks
Strategy Variations			
D. Weekly Sweeps	50%	21	21 Weeks
E. Biweekly Sweeps (every 2 weeks)	100%	11	21 Weeks
F. Sweeps Twice a Week	100%	41	21 Weeks
G. Weekly Sweeps for First 6 Weeks of Peak Transmission Season	100%	7	6 Weeks
H. Biweekly Sweeps for First 12 Weeks of Peak Transmission Season	100%	7	12 Weeks
I. Combining Weekly and Biweekly Sweeps: Weekly from Week 5 to Week 11, Biweekly at Weeks 1, 3, 12, 14, 16, 18, and 20	100%	14	20 Weeks
J. Combining weekly and biweekly sweeps: Weekly from Week 5 to Week 11, Biweekly at Weeks 3, 12, 14, and 16	100%	11	13 Weeks

PROCCM = proactive community case management.

### No patient or public involvement.

There was no patient or public involvement in the study design of this research, the interpretation of the results, or the writing or editing of this document.

## RESULTS

**Figure 3.**
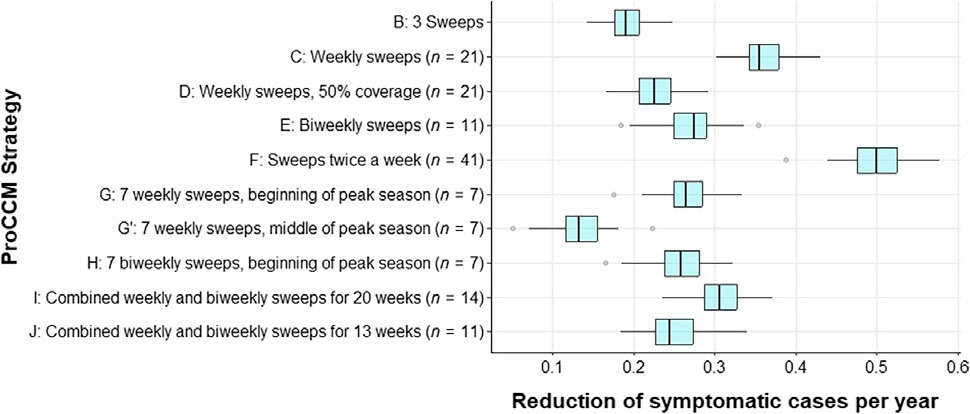
Box plot showing the reduction of symptomatic cases (from 0.0 to 1.0) per year compared with Strategy A (no sweeps) for strategies reported in Table 1. G and G′ refer to sweeps during the first and middle 6 weeks of peak transmission season, respectively. H refers to biweekly sweeps during the first 12 weeks of peak transmission season. Strategy F gives the maximum reduction but with maximum effort (two sweeps/week for the duration of the season). PROCCM = proactive community case management.

**Figure 4.**
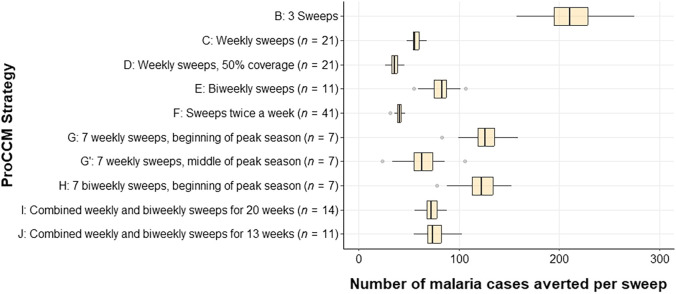
Box plot showing the number of symptomatic infection cases averted per sweep (compared with no sweeps) under various strategies reported in Table 1. G and G′ refer to sweeps during the first and middle 6 weeks of peak transmission season, respectively. H refers to biweekly sweeps during the first 12 weeks of peak transmission season. PROCCM = proactive community case management.

**Figure 5.**
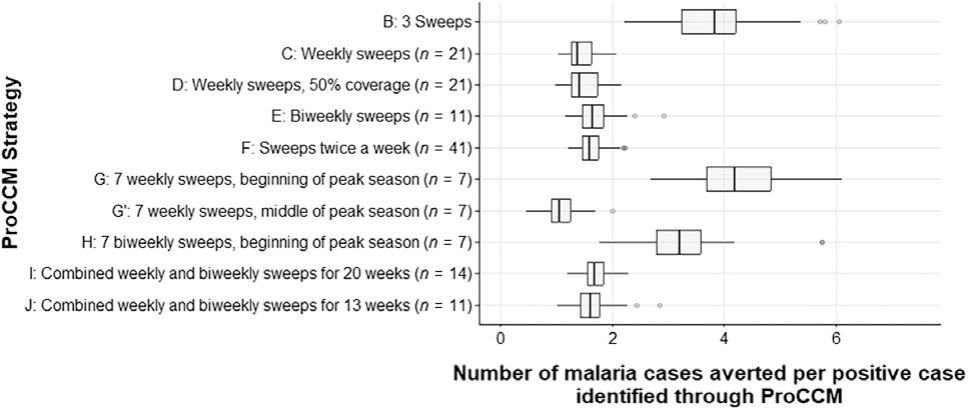
Box plot showing the number of symptomatic infection cases averted per positive case identified per sweep (compared with no sweeps) under various strategies reported in Table 1. G and G′ refer to sweeps during the first and middle 6 weeks of peak transmission season, respectively. H refers to biweekly sweeps during the first 12 weeks of peak transmission season. PROCCM = proactive community case management.

Depending on the frequency and duration of sweeps, the reduction in symptomatic malaria cases varied from 19.3% for three sweeps to 50.1% for biweekly sweeps strategies. Figure 3 shows the year-round percentage reduction (compared with the base case of no sweeps [Strategy A]; 1.0 = 100% reduction) for symptomatic malaria cases, and Figure 4 shows the number of symptomatic malaria cases averted per sweep (for all ages, compared with Strategy A) under the different sweep strategies defined in Table 1. Figure 5 reports the number of symptomatic cases averted per positive case identified through ProCCM. Simulation results for each strategy are reported in Supplemental Appendix, Section 3, Supplemental Table 8.

In Strategy B from Table 1, shown in Figure 1, we observe that three sweeps at the beginning, middle, and end of the malaria peak season reduces the number of symptomatic infection cases per year by 19.3%, the lowest of the sweep scenarios modeled apart from Strategy G′ which is a variation of Strategy G with 7 weekly sweeps during the middle rather than beginning of peak season. Symptomatic malaria cases significantly decline right after each ProCCM sweep. Infected patients who are identified during a sweep receive treatment and, although not fully recovered immediately, are removed from the set of symptomatic patients. This number returns to the same level as when no sweeps were conducted after 7–10 days. Because of the high infection prevalence within the mosquito population during the peak season compared with nonpeak season, identifying and treating infected humans at too low of a frequency would not effectively control malaria infection at the community level. Conducting weekly sweeps for 21 consecutive weeks (Strategy C of Table 1) prolongs the effect of ProCCM and provides a much more promising result with a 36.3% reduction of symptomatic cases per year. Moreover, the peak weekly number of symptomatic malaria infections is less than half compared with no intervention. When Strategy I (Table 1) is adopted, combining weekly and biweekly sweeps as described for a total of 14 sweeps during peak season, there is a 30.5% reduction in symptomatic infection cases per year.

Based on our simulation results, the most obvious factor influencing the ProCCM outcome is sweep frequency. However, the relationship between level of reduction in malaria infection cases and sweep frequency is not linear, as shown in Supplemental Appendix, Section 3, Supplemental Table 8. Biweekly sweeps during peak season, 11 sweeps per year, could reduce malaria infection cases by 26.9% per year. If conducted weekly during peak season, the infection cases are reduced by 36.3%. This number can be increased even further to 50.3% by doubling the sweep frequency again to twice weekly (Strategy F). Higher sweep frequency results in more effective malaria infection control, as one would expect.

Decreasing sweep coverage from 100% to 95% does not have a big impact (Supplemental Appendix, Section 3, Supplemental Tables 9 and 10). However, the impact is amplified as coverage decreases further. In the extreme case, if coverage is only 50% (Strategy D), weekly sweeps requires more RDTs and results in more infections than biweekly sweeps with full coverage (Strategy E).

We performed sensitivity analyses to determine the impact of variations to the treatment seeking rate parameters incorporated in the model. We found that the treatment-seeking rate at severe condition had less impact on the number of symptomatic cases than the treatment-seeking rate at mild condition (Supplemental Appendix, Section 3, Supplemental Figures 11 and 12). With a higher treatment-seeking rate at mild conditions, we saw large drops in both the number of symptomatic cases and the cases detected by sweeps.

## DISCUSSION

Using an agent-based simulation model, we predict that with optimized deployment ProCCM could be very effective in reducing symptomatic confirmed malaria infection at the community level in this setting with moderate, highly seasonal transmission. Annual symptomatic malaria cases could be reduced by as much as 36.3% by conducting weekly sweeps during the peak transmission season. Early start, more sweeps, and shorter delays between sweeps (preferably weekly) are critical factors in improving the ProCCM outcome, although these all increase costs. For example, Strategy F had the largest reduction in symptomatic cases (Figure 3) but the number of cases averted per sweep was low (Figure 4), implying that the benefit of that strategy may not outweigh the operational cost of performing two sweeps per week. Figure 5 highlights the importance of starting sweeps early and intensively, as Strategies G and H had large numbers of cases averted per positive case identified relative to other strategies. Considering these factors, we proposed an alternative strategy of varying sweeps between weekly and every other week during peak season (with weekly sweeps in midseason and sweeps every other week at either end) that could reach a similar level of symptomatic malaria reduction compared to weekly sweeps while reducing the intervention inputs by 33.3%.

The effectiveness of ProCCM in reducing symptomatic malaria infection in this setting can be explained on the individual and community levels. Early detection may allow for identification of infection at a lower parasite density, which could lead to a shorter recovery time after treatment, less chance of treatment failure, and a lower mortality rate.^10^ Furthermore, an infected human who has not yet developed gametocytemia at the time of treatment is less likely to spread the infection to mosquitoes during and after treatment.^21^ By reducing the chance of infecting uninfected mosquitos and shortening the duration of infectiousness, malaria prevalence may be reduced at the community level. The simulation results also confirm such conclusions. The year-round symptomatic cases of all ages were constantly increasing with delayed starting time in our comparison of seven consecutive weekly and biweekly sweeps with different starting dates (Supplemental Appendix, Section 3, Supplemental Tables 12 and 13). In theory, if ProCCM could detect, treat, and cure all infected humans before they became gametocytemic (infectious to mosquitoes), the transmission between human and mosquito populations would eventually end once all asymptomatic individuals recovered. If it is not possible to conduct sweeps starting at the beginning of the transmission season and continuing throughout the season, simulation results indicate that the effective starting point for intervention is within the first 2–3 weeks after the beginning of transmission season if only seven consecutive sweeps are conducted. More details are included in the Supplemental Appendix, Section 3.

In addition to starting time, our simulation results suggest that sweep coverage and more sweeps in longer duration are other critical factors for ProCCM in this setting. A longer intervention period is beneficial, as one would expect. Our simulation model showed a significant increasing trend in the mosquito population 6 weeks prior to the start of peak season (early July in Senegal, around week 27), which provides more opportunities for malaria transmission. Thus, early intervention would decrease the infection level later in the transmission season.

Because the mosquito population is significantly smaller outside the peak season, the effect of ProCCM sweeps during this time is predicted to be limited. After the peak season, as there are no new emerging mosquitos joining the adult population, transmission wanes. Comparing the results from Strategy E and Strategy H, biweekly sweeps after the first 12 weeks of peak season will not reduce the total number of symptomatic cases and thus would not be cost-efficient.

Assuming ProCCM implementation cost is directly related to the number of sweeps conducted, Strategy I appears potentially most cost-efficient. Compared with weekly sweeps, Strategy I from Table 1 (where biweekly and weekly sweeps were combined with higher frequency during midpeak season) resulted in only 9.1% increases in the total number of year-round symptomatic malaria infection cases, but resulted in reductions of 33.3% of the cost of annual weekly sweeps. Compared with Strategy E (biweekly sweeps) and Strategy H (biweekly sweeps for the first 12 weeks), Strategy J (where a combination of 11 biweekly and weekly sweeps were conducted with higher frequency during midpeak season) missed the opportunity to control the parasite density in the beginning of the transmission season and therefore did not improve outcomes. For a fixed number of sweeps with fixed cost, early action will result in the best outcome. Maintaining a high coverage per sweep (90% or more) will be important for a successful ProCCM implementation.

It is worth noting that the effectiveness of ProCCM depends largely on the local residents’ treatment-seeking behavior (e.g., with a higher treatment-seeking rate, more symptomatic cases will seek care prior to being identified during a sweep, making the sweep less effective). Despite 60% of severe case patients actively seeking treatment between weekly sweeps,^11^ we still need the help of ProCCM to discover the remaining patients and control malaria transmission. ProCCM contributes significantly to increasing the treatment-seeking rate, as documented in other studies of active case detection.^24^^,^^25^

Our study highlights the benefits of ProCCM, with particular emphasis on starting the intervention early and with a sufficient number of sweeps and coverage. In a ProCCM pilot study conducted in Mali, after several years of continuous sweeps combined with providing more convenient treatment access, the number of febrile cases among children under 5 years declined significantly.^26^ In contrast, in Madagascar, the ProCCM intervention had less impact on reducing symptomatic malaria cases than in Senegal owing to relatively insufficient sweeps frequency and coverage^15^ and delayed starting time after the transmission peak,^27^ similar to Strategy G′.

### Limitations.

Many community case management models include treatment of diarrhea and pneumonia for children under 5 years, screening for malnutrition, and other services. The model described herein did not account for benefits derived from diagnosis and treatment of these often comorbid conditions.

There are parameters in our human infection transition model that were estimated within certain published ranges. We used 50 replications for each simulation case to minimize the variance. In addition, we adopted parameters that fit our model the best and provided the closest result to existing data in Linn et al.^11^ We may not have perfectly captured the whole picture of malaria transmission owing to simplifications regarding immunity and chronic asymptomatic infection. However, the primary purpose of this paper was not to provide a comprehensive transmission model but to focus on the deployment of ProCCM while preserving the essence of malaria transmission. To test the robustness of our conclusions, we ran simulations with varying values for the environment’s carrying capacity. The results show that although magnitudes of reductions of symptomatic cases may change, the conclusions and comparisons between strategies still hold. More details are included in Supplemental Appendix, Section 4.

This model was built using data from a very specific context, with highly seasonal, relatively moderate transmission in the Sahel, in which ProCCM appeared to be highly effective at reducing malaria cases. The model does not predict outcomes in areas of different transmission seasonality and intensity where input variables such as temperature, precipitation, and human characteristics are different. When applying our methods to other regions or climates, these parameters are subject to change or re-estimation, but with some calibration the model could be adapted to other malaria-endemic regions. If the transmission vectors are mosquitos of other species, further effort will be required to verify the applicability of our mosquito dynamic model before simulation.

Our results suggest the importance of early intervention, and for simplicity, we assumed that the cost of each strategy was proportional to the number of sweeps. However, such an assumption may not hold in real-life scenarios, and a more detailed cost-efficiency evaluation is recommended before an intervention strategy is implemented.

## CONCLUSION

In this paper, we developed an agent-based simulation to model the impact of various ProCCM strategies on symptomatic malaria cases in a sub-Saharan African country with moderate and highly seasonal malaria transmission. We validated our model using available data from Linn et al.^11^ Our model suggests that with a well-designed deployment strategy, ProCCM can be highly effective at reducing symptomatic malaria during peak transmission season. Under a highly seasonal transmission setting, trade-off studies showed that starting the sweeps 5 weeks before peak transmission season and continuing for a longer duration at a higher frequency would increase the impact of ProCCM, though with diminishing returns. To reduce the number of malaria infection cases by 30% per year, sweeps would need to be conducted for the first 12 weeks during the transmission season, with at least a fortnightly frequency in this setting. Our model is flexible and allows decision-makers to evaluate alternative implementation strategies incorporating combinations of sweeps that occur weekly or at lesser frequencies, depending on the time of the year and available budget.

## Supplemental Materials

10.4269/ajtmh.23-0844 Supplemental Materials
