# Supplementary material for: Modeling the Impact of Proactive Community Case Management on Reducing Confirmed Malaria Cases in Sub-Saharan African Countries
Source: Am J Trop Med Hyg. 2024 Jul 9;111(3):490–7. doi: 10.4269/ajtmh.23-0844 (PMC11376187; doi:10.4269/ajtmh.23-0844)
Supplement: Supplemental Materials [file tpmd230844.SD1.pdf]

Appendix

Section 1: Data

We estimate the treatment seeking rate for fever in children under 5 to be the same as that of older children and adults[1] and 30% of the population aged over five have immunity against malaria[2] at the beginning of the simulation (Table S1).

To capture the impact of seasonality on parasitemia and hence transmission patterns, we use temperature and precipitation data from 2010 to 2013 obtained from National Climactic Data Center Data Online[3] (Figure S1).

While ITN (insecticide treated nets) coverage data is shown, the data are not used in the model and net coverage is not directly modeled in the study. We looked at the final symptomatic cases, which we agree is also a result of the net coverage. However, because the net coverage in the intervention and comparison groups are so similar, including this in the model would not create large impacts to the results with respect to comparison of different strategies. Excluding nets from the analysis gives us a conservative relative estimate of the benefit of ProCCM: if net coverage was lower, then the benefit from ProCCM would be higher. Future study could investigate the impact of ProACT under varying levels of net coverage.

The malaria transmission models proposed in this paper are built comprehensively on existing literature.

Table S1 Human characteristics data for the simulation model

Setting	Three Sweeps (Comparison Group)	Weekly Sweeps (Intervention Group)	Reference
Population	4747, within 15 villages	3762, within 14 villages	4
Net coverage	100%	98.40%	
Symptomatic Malaria Prevalence [%] (on July 8th, Sep 23rd and Nov 25th)	[1.58, 2.97, 3.35]	[1.88, 1.15, 0.21]	
Treatment seeking rate	40%	37%	
Children under 5	15%	15%	Estimated based on [1]
Immune human	30%	30%	
Birth rate	33.4/1000 ppl per year	33.4/1000 ppl per year	5
Natural Death rate	5.9/1000 ppl per year	5.9/1000 ppl per year	

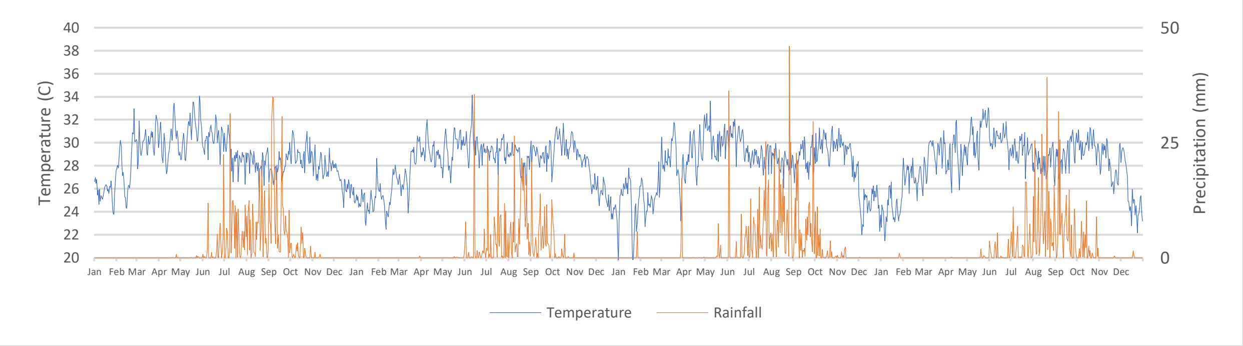

Figure S1 Temperature and precipitation data in Senegal from 2010 to 2013

Section 2: Model Development

Section 2.1: Simulation Model of the Mosquito Population

To model the dynamics of the mosquito population, we use the general framework proposed in Cailly et al.[6] for *Anopheles*, and Tran at al[7] for *Aedes albopictus*. Table S2 and Figure S2 depict the stages in the life cycle of mosquitos: aquatic stages (E, eggs; L, Larvae; P, pupae), emerging adult stage (A_{em}), nulliparous stages (A_{1h}, A_{1g}, A_{1o}) and parous stages (A_{2h}, A_{2g}, A_{2o}). Note that only the female mosquitoes are represented in the adult stage.

Table S2 Mosquito life cycle stages

Stage	Description
E	Eggs
L	Larvae
P	Pupae
A_{em}	Emerging adults
A_{1h}	Host-seeking nulliparous
A_{1g}	Nulliparous engorged
A_{1o}	Nulliparous seeking oviposition sites
A_{2h}	Host-seeking parous
A_{2g}	Parous engorged
A_{2o}	Parous seeking oviposition sites

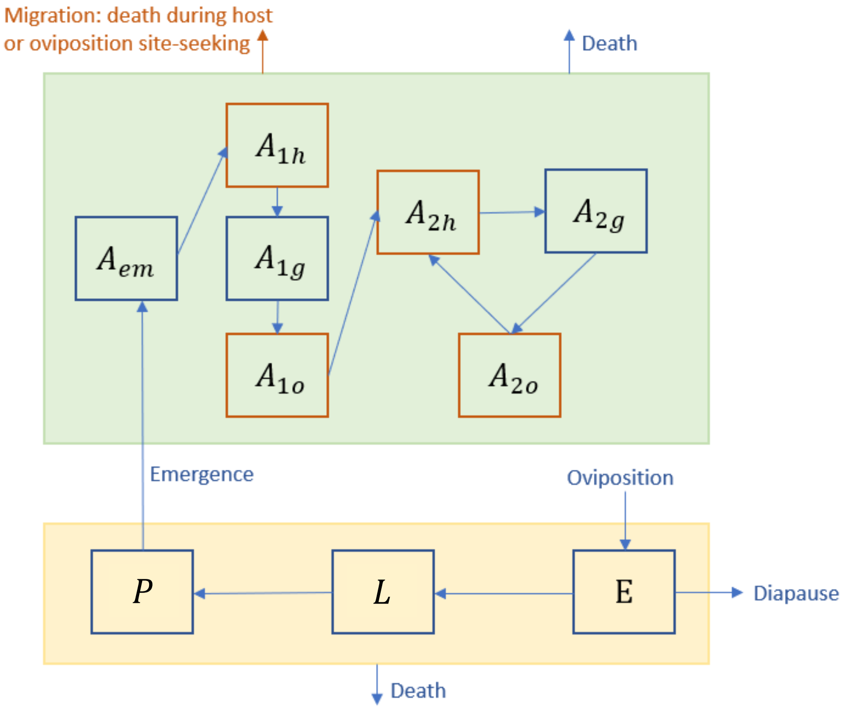

Figure S2 Mosquito population dynamic model representing the stages, and transitions between stages, in the life cycle of mosquitos.

The duration of each stage in the mosquito life cycle depends on several factors, such as temperature and water availability (precipitation). Temperature impacts the mortality and transition rates of larvae, pupae and adults, while precipitation impacts the environment's carry capacity of aquatic stages, increasing the number of breeding sites available for mosquitos. For example, the climate in Senegal is tropical with high

temperatures all year round and rainy season from May through November. The high transmission season starts in early July and ends in late October. We divide a year into favorable season and diapause period, where eggs stop hatching until the next favorable season when they hatch if they are immerged in water.

The mosquito population dynamic model is based on a system of ordinary differential equations (Equation (1)); these equations model how the mosquito population behaves over time, taking into account factors such as mortality rates at different stages and the temperature required for egg development. The parameters and functions are defined in Table S3 and Table S4. The adult female mosquito population (N_v) is divided into three groups: uninfected mosquitos (S_v), infected but not yet infectious mosquitos (C_v), and infectious mosquitos (I_v), i.e., $N_v = S_v + C_v + I_v$. T(t) and P(t) represent the daily mean temperature in Celsius and precipitation in millimeters, respectively, on day t. $\mathrm{P_{norm}}(t)$ is defined as the rainfall amount summed over a two week period and normalized afterwards.

Equation (1): mosquito population dynamic model

$$\begin{cases} \dot{E} = \gamma_{Ao}(\beta_1 A_{1o} + \beta_2 A_{2o}) - (\mu_E + z f_E)E \\ \dot{L} = (z f_E)E - [m_L\left(1 + \frac{L}{k_L}\right) + f_L]L \\ \dot{P} = f_L L - [m_P + f_P]P \\ \dot{A_{em}} = f_P P \sigma \exp\left[-\mu_{em}\left(1 + \frac{P}{k_p}\right)\right] - [m_A + \gamma_{A_{em}}]A_{em} \\ \dot{A_{1h}} = \gamma_{A_{em}} A_{em} - (m_A + \mu_r + \gamma_{A_h})A_{1h} \\ \dot{A_{1g}} = \gamma_{A_h} A_{1h} - \left(m_A + f_{A_g}\right) A_{1g} \\ \dot{A_{1o}} = f_{A_g} A_{1g} - (m_A + \mu_r + \gamma_{Ao})A_{1o} \\ \dot{A_{2h}} = \gamma_{A_o}(A_{1o} + A_{2o}) - (m_A + \mu_r + \gamma_{A_h})A_{2h} \\ \dot{A_{2g}} = \gamma_{A_h} A_{2h} - \left(m_A + f_{A_g}\right) A_{2g} \\ \dot{A_{2o}} = f_{A_g} A_{2g} - (m_A + \mu_r + \gamma_{A_{2o}})A_{2o} \end{cases} \quad (1)$$

where z = 0 during diapause and 1 otherwise.

Table S3 Parameters for mosquito population dynamic model

Parameters	Definition	Value	Reference
β_1	Number of eggs laid by ovipositing nulliparous females (per female)	100	
β_2	Number of eggs laid by ovipositing parous females (per female)	150	
κ_L	Standard environment carrying capacity for larvae (larvae ha^{-1})	350,000	
κ_P	Standard environment carrying capacity for pupae (pupae ha^{-1})	350,000	
σ	Sex-ratio at emergence	0.5	
μ_E	Egg mortality rate (day^{-1})	0.05	
μ_L	Minimum larva mortality rate (day^{-1})	0.08	
μ_P	Minimum pupa mortality rate (day^{-1})	0.03	
μ_{em}	Mortality rate during adult emergence (day^{-1})	0.1	
μ_A	Minimum adult mortality rate (day^{-1})	0.02	6,7
μ_r	Adult mortality rate related to seeking behavior (day^{-1})	0.08	
T_E	Minimal temperature needed for egg development (C^o)	10.4	
TDD_E	Total number of degree-day necessary for egg development (C^o)	110	
T_{Ag}	Minimal temperature needed for egg maturation (C^o)	10	
TDD_{Ag}	Total number of degree-day necessary for egg maturation (C^o)	77	
$\gamma_{A_{em}}$	Development rate of emerging adults (day^{-1})	0.4	
γ_{A_h}	Transition rate from host-seeking to engorges adults (day^{-1})	0.2	
γ_{Ao}	Transition rate from oviposition site-seeking to host-seeking adults (day^{-1})	0.2	
t_{start}	Start of the season favorable for mosquito growth	10 March	Adjustable by region
t_{end}	End of the season favorable for mosquito growth	30 Oct	

Table S4 Functions incorporated in mosquito population dynamic model

Function	Definition	Expression	Reference
f_E	Transition function from egg to larvae	$f_E(t) = \begin{cases} \frac{T(t) - T_E}{TDD_E}, \text{if } T(t) > T_E \\ 0 \end{cases}$	
f_L	Transition function from larvae to pupae	$f_L(t) = -0.0007T(t)^2 + 0.0392T(t) - 0.3911$	
f_P	Transition function from pupae to emerging adult	$f_P(t) = 0.0008T(t)^2 - 0.0051T(t) + 0.0319$	
f_{Ag}	Transition function from engorged stage to oviposition-site-seeking stage	$f_{A_g}(t) = \begin{cases} \frac{T(t) - T_{A_g}}{TDD_{A_g}}, \text{if } T(t) > T_{A_g} \\ 0 \end{cases}$	
m_L	Larva mortality rate (day^{-1})	$m_L(t) = \exp\left(-\frac{T(t)}{2}\right) + \mu_L$	6,7
m_P	Pupa mortality rate (day^{-1})	$m_P(t) = \exp\left(-\frac{T(t)}{2}\right) + \mu_P$	
m_A	Adult mortality rate (day^{-1})	$m_A(t) = \max(u_A, 0.0441 + 0.00217T(t))$	
k_L	Environment carrying capacity of larvae (ha^{-1})	$k_L(t) = \kappa_L(P_{norm}(t) + 1)$	
k_P	Environment carrying capacity of pupae (ha^{-1})	$k_P(t) = \kappa_P(P_{norm}(t) + 1)$	
$B(T(t))$	Mosquito biting rate (day^{-1})	$0.000203T(t)(T(t) - 11.7)\sqrt{}(42.3 - T(t))$	

Based on the model proposed, we generated an adult host seeking mosquito $(A_{1h} + A_{2h})$ population for 4 years, shown in Figure S3. The initial mosquito population contains 1,000,000 eggs, and is used for the start of each new season. The simulation includes a warm-up period of one year, which is not included in the plot.

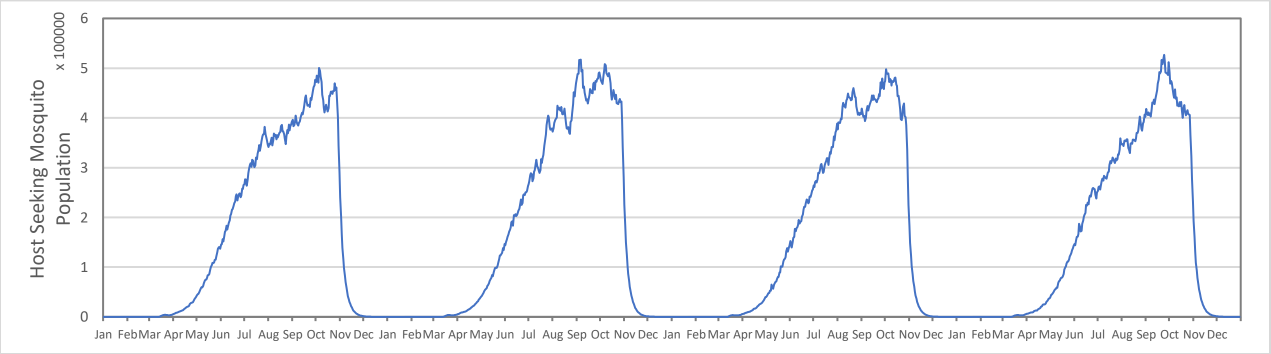

Figure S3 Host-seeking mosquito population in Saraya from 2010 to 2013 using simulation

Section 2.2. Model Parameters for Human Infection

Our simulation model is based on a modification of Winskill et al[8] capturing the same progression process between human infection states while making a few parsimonious changes such as classifying humans into categories based on age, and integrating immunity, which means a human with “anti-parasite” immunity that confers protection against high-density parasitemia and the attendant risk of severe disease, also has “anti-disease” immunity that lowers the chances of developing malaria from an infected mosquito. Both types of immunity tend to develop in parallel with increasing age, and therefore can be integrated together[9]. Also, although Rodriguez-Barraquer et al. refer to “anti-disease” immunity as a type of immunity, because it mostly distinguishes itself by modulating host response to parasitemia (i.e., symptoms), and not response to the parasites themselves, we think of the second “type” more as

immune system modulation and not immunity per se. Furthermore, the authors mention that "anti-disease immunity" is not completely understood, and since it can or cannot have a concomitant effect on parasitemia – which is what we are interested in for the dynamic model – it is more conservative to model the immunity effects as one. Therefore, in the following content, we will not differentiate two types of immunity. These modifications simplify the age-based simulation model while still capturing the essence of the malaria transmission process.

We first divide the human population into two groups: children under 5 (C) and adults (A). The transition processes between disease states follows the same patterns for both groups, but with different parameters.

At a given time, a human (child or adult) is in one of the following states based on his/her infectious condition: uninfected non-immune (S_h), infected but not yet infectious non-immune (C_h), infectious non-immune (I_h), uninfected immune (SR_h), infected but not yet infectious immune (CR_h), infectious immune (IR_h).

A human infection begins with a successful contact by an infectious mosquito. In an infected human body, the malaria parasite undergoes a pre-erythrocytic liver stage first, which typically lasts for 1–2 weeks, before the onset of the blood stage. During the blood stage, the sexual form of the malaria parasite, the gametocyte, is produced and thus the infected human becomes infectious to mosquitoes[10]. The duration from a human being infected by a mosquito to him/her being infectious to mosquitoes is defined as the incubation period.

After a successful infection by a mosquito, the infected human also goes through (some of) the following phases, based on the severity of their symptoms:

Infection →Asymptomatic Parasitemia (A_h) → Uncomplicated Illness (M_h^m) → Severe Malaria (M_h^S) →Death[11].

An infected human first goes through asymptomatic parasitemia phase (A_h), which lasts between 6 to 14 days[12], in which there are no symptoms. After this, he/she begins to show mild (uncomplicated) illness

symptoms, including fever, chills, headaches, diaphoresis, etc. If not actively seeking treatment, a human in the uncomplicated illness phase could go into the severe malaria phase after 5-7 days for adults and 1-2 days for children under 5, as the parasite density accumulates within his/her body[10] . Since the duration of the incubation state doesn't necessarily coincide with the asymptomatic parasitemia phase, an infected human can become infectious either at the end of the asymptomatic parasitemia phase or at the beginning of the uncomplicated illness phase.

We further differentiate the level of human infectiousness depending on where a human is among the phases of the infected state. As gametocytes in *P. falciparum* infections arise from asexual parasites (i.e., merozoites), there could be a positive correlation between the density of asexual and sexual parasite (gametocytes)[2] . In nonimmune individuals, hyperparasitemia (>5% parasitemia or >250 000 parasites/ul) is generally associated with severe disease[12]. Research also suggests that the level of infectiousness of a human is a concave increasing function of female gametocyte density[13]. Thus, we assume that the human level of infectiousness, $\mathrm{i_H}$, linearly increases from 0 to 1 during the uncomplicated illness phase, i.e.,

$$\mathrm{i_H} = \mathrm{t}/\mathrm{T_{in}},$$

where t is how long an infected human has been in the uncomplicated illness phase, and $\mathrm{T_{in}}$ is the total duration of the uncomplicated illness phase. The human's infectiousness level is highest, i.e., $\mathrm{i_H} = 1$, during the severe malaria phase. State and transition parameters for the human infection dynamic model are provided in Tables S5-S7.

Table S5 State parameters representing the duration between phases of infection for the human infection dynamic model

States	Explanation	Duration [Day] T_X	Notes & References
T_1	Infected non-immune human → Non-immune human with mild symptoms	6- 14days	[12]
T_2	Non-immune human with mild symptoms → Non-immune human with severe symptoms	Adult: 5-7 days<br>Children: 1-2 days	[10]
T_3	Non-immune human with mild symptoms → (Recovery) Uninfected non-immune/immune human	Mean: 6.6 days/ 14.5 days	[14]
T_4	Non-immune human with severe symptoms → (Recovery) Uninfected non-immune/immune human	7-35 days	[12,15]
T_5	Infected immune human → Immune human with mild symptoms	7-30days	[16,17]
T_6	Infected immune human → (Recovery) Uninfected non-immune/immune human	14-120 days	Estimated; uninformative, uniform Bayesian prior
T_7	Immune human with mild symptoms → (Recovery) Uninfected non-immune/immune human	7-90 days	[14]
T_i	Infected human → Infectious human	7-15 days after initial infection	[10]

Table S6 Transition parameters representing the probability of moving between states in the human infection dynamic model

Transition Arcs	Meaning	Parameters needed
$\mathbf{S_h} \rightarrow \boldsymbol{A_h}$	Non-immune human infected	$c_h^n, B(T)$
$\mathbf{SR_h} \rightarrow \boldsymbol{AR_h}$	Immune human infected	$c_h^i, B(T)$
$\mathbf{A_h} \rightarrow \boldsymbol{M_h^m}$	Non-immune human showing symptoms after incubation	T_1
$\mathbf{AR_h} \rightarrow \boldsymbol{MR_h}$	Immune human showing symptoms after incubation	T_5
$\boldsymbol{M_h^m} \rightarrow \boldsymbol{M_h^s}$	Non-immune individual with mild malaria progress to severe malaria	T_2
$\boldsymbol{M_h^m} \rightarrow \boldsymbol{D_h}$	Non-immune individual with mild malaria deceased	P_d^m
$\boldsymbol{M_h^s} \rightarrow \boldsymbol{D_h}$	Non-immune individual with severe malaria deceased	P_d^S
$\mathbf{AR_h} \rightarrow \boldsymbol{SR_h}$	Immune human recovery without showing symptoms	$T_6, \mathbf{P_s}$
$\boldsymbol{M_h^m} \rightarrow \boldsymbol{S_h}$	Non-immune human recovery with mild condition without gaining immunity	$T_3, P_{sc}, P_{st}^m, \boldsymbol{P_{gi}}$
$\boldsymbol{M_h^s} \rightarrow \boldsymbol{S_h}$	Non-immune human recovery with severe condition without gaining immunity	T_5, P_{sc}, P_{st}^s
$\boldsymbol{M_h^m} \rightarrow \boldsymbol{SR_h}$	Non-immune human recovery with mild condition while gaining immunity	$T_4, P_{sc}, P_{st}^m, P_{gi}$
$\boldsymbol{M_h^s} \rightarrow \boldsymbol{SR_h}$	Non-immune human recovery with severe condition while gaining immunity	$T_5, P_{sc}, P_{st}^s, P_{gi}$
$\mathbf{MR_h} \rightarrow \boldsymbol{SR_h}$	Immune human with mild condition recovery without losing immunity	T_6, P_s, P_{li}
$\mathbf{MR_h} \rightarrow \boldsymbol{S_h}$	Immune human with mild condition recovery while losing immunity	T_6, P_s, P_{li}
$\boldsymbol{\lambda_h}$	Natural birth rate	33.4/1000
$\mathbf{f_h}$	Death rate of natural causes (uniform in all stages)	5.9/1000

Table S7 Parameter values for the human infection dynamic model

Parameter	Definition	Data	Reference
$\mathbf{c}_h^n$	Infected non-immune human to mosquito transmission efficiency	0.5	Estimated based on [18]
$\mathbf{c}_h^i$	Infected immune human to mosquito transmission efficiency	0.25	
$\mathbf{c}_\mathbf{v}^\mathbf{n}$	Infected mosquito to uninfected non-immune human transmission efficiency	0.5	
$\mathbf{c}_\mathbf{v}^i$	Infected mosquito to uninfected immune human transmission efficiency	0.25	
P_r^m	Probability of self-recovery from mild symptom [adult, child]	[0.2, 0.1]	[19]
P_r^S	Probability of self-recovery from severe symptom [adult, child]	[0.01, 0]	[11]
P_{st}^m	Probability of seeking treatment at mild condition	0.25	Estimated based on [1]
P_{st}^s	probability of seeking treatment at severe condition	0.60	[19]
P_d^m	Mortality at mild infectious stage [adult, child]	[0.01,0.04]	[20]
P_d	Base mortality at severe infectious stage [adult, child]	[0.10, 0.20]	
P_d^S	Mortality at severe infectious stage with time	linearly increase from P_d to 1	[11,21]
P_{gi}	Probability of gaining immunity after recovery	0.3	Estimated based on [22]
P_{li}	Probability of losing immunity after recovery	0.05	
P_{tf}	Probability of treatment failure	0.05	[12]
P_{sc}	ProCCM sweep coverage	0.80-1.00	Estimated based on [23]
$\mathbf{P_s}$	Probability of immune infectious human showing symptoms	0.1	[2]

Section 2.3 Interaction between Human and Mosquito Populations

An uninfected clean non-immune human is susceptible to malaria and could be infected (with probability I_v) after a bite from an infectious mosquito. We assume that every human has equal chance of being contacted by any mosquito[24] and children under 5 years old do not have immunity and thus, always show symptoms if infected ($\boldsymbol{P_{gi} = 0}$)[22]. Although recent literature has shown that these assumptions are not quite realistic, particularly with regard to non-uniform infectivity[25,26] for simplicity of the hybrid model (which models mosquitos with simple Ordinary Differential Equations and humans with more complex agent-based models) we have let them stand in the current model, with the understanding that transmission results are not as conservative as they could be.

We adopt the idea of effective contact ratio that determines daily numbers of newly infected humans (NI_h) and newly infected mosquitos (NI_v)[27] and incorporate the individual infectiousness level, $\mathrm{i_H}(t) \in [0,1]$.

The adjusted effective contact ratio equations are:

$$NI_h = \left(\frac{C_v B(T) I_v}{N_h}\right)(P_{S_h} + P_{SR_h}) \tag{2}$$

$$NI_v = \sum_{inv \in P_{I_h}} \left(\frac{C_h B(T) i_{inv}}{N_h}\right) S_v \; + \sum_{inv \in P_{IR_h}} \left(\frac{C_h B(T) i_{inv}}{N_h}\right) S_v \tag{3}$$

where $\mathrm{c_v}, c_h$ are the successful biting rates, B(T) is the mosquito biting rate determined by mean daily temperature, N_h is the total number of humans in certain age group, i_{inv} is the individual infectiousness level and P_x is the total number of humans in a certain infectious condition, e.g. P_{I_A} is the total number of infectious non-immune adults. Mandel et al. [18] and Filipe et al.[28] report values for successful biting rates in the range 0.2-0.5, but do not distinguish if humans are immune or naïve. The parameter values we have chosen are an educated guess based on these references and the fact that, because humans are immune or naïve (for which we use the higher 0.5 value), we are using the lower end of the range.

Section 2.4 Treatment and Residual Parasitemia

A human with uncomplicated malaria or severe malaria has the possibilities of "self-recovering", e.g., self-treatment, seeing a traditional healer, taking herbal medication[19] or simply recovering as the immune system responds, although the chance is small in the case of severe malaria. If a symptomatic human seeks treatment, at any stage after developing symptoms, we assume that he/she will receive artemisinin-based combination treatments (ACTs) for 3 days[10], and the recovery process starts immediately after the first day[29]. Thus, the human infectiousness level would stop increasing as soon as the treatment begins. Possibilities of treatment failure ($\boldsymbol{P_{tf}}$) and mortality depends on the how long one has been infected as well

as the severity of his/her symptoms. After the completion of treatment, some humans (50%) would be parasite free and the others would have residual parasitemia [31]. Humans with residual parasitemia have higher gametocyte density after treatments, and longer gametocyte carriage durations compared with those who are parasite free after ACTs[14]. In this model we assume that, after ACTs, a human preserves 50% to 75% of the infectiousness before treatment, and this level linearly decreases until the end of gametocyte carriage duration.

Section 2.5 Acquired Immunity against Malaria

After recovery, a non-immune human can gain immunity with a certain probability ($\boldsymbol{P_{gi}}$)[22]. An immune human will become infected after a successful bite from an infected mosquito with a smaller chance[16]. After being infected, the immune human will then go through the asymptomatic parasitemia phase with a longer duration[30] . It is shown that with immunity, parasite density within an infected human body would be lower. Thus, an immune human with asymptomatic parasitemia will have a much smaller possibility of showing symptoms, move to the uncomplicated malaria phase, and will be extremely unlikely to eventually progress into the severe malaria phase[2]. The majority of the immune humans with asymptomatic parasitemia will self-recover. Some who develop symptoms after the asymptomatic parasitemia phase may recover if they seek treatments. Losing immunity after recovery (P_{ii}) is also very unlikely under consistent heavy malaria exposure[27].

Newborns begin their lives as uninfected non-immune humans. All humans are susceptible to natural death at a fixed rate. Traditionally, an infected human would only actively seek treatment ($\boldsymbol{P_{st}}$) if he/she developed symptoms. Treatment-seeking possibilities are different between humans in the adult group and children. Those who don't seek treatment will keep infecting uninfected mosquitos. ProCCM could actively detect infected humans with symptoms, give them treatments and terminate the infection loop.

All the states are listed in Table S5. The duration range for each state is adopted from corresponding literature. We assume that all distributions are uniform within the given ranges. The transition diagram for our human infection model is shown in Figure S4.

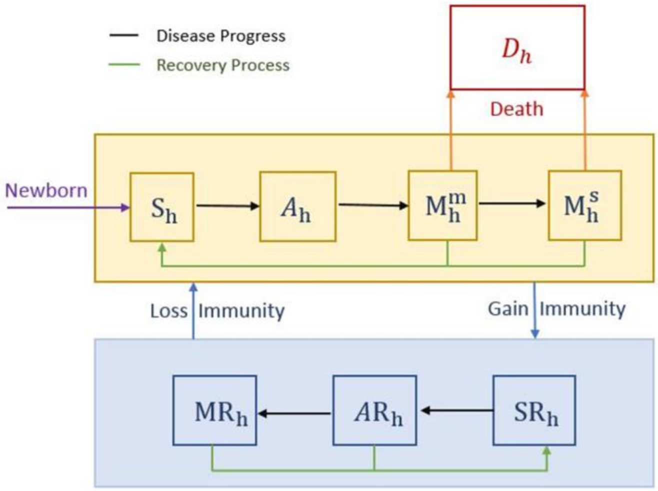

Figure S4 Human infection transition graph representing the stages, and transitions between stages, of human infections.

The parameters used in this simulation model are listed in Table S6 and Table S7.

Section 3. Model Validation, Experimental Results, and Sensitivity Analysis

In Linn et al.,[4] 1,669 symptomatic infection cases are documented in the pilot intervention group from July to November, when 89% of the weekly sweeps are conducted and the total population is 3,762. With all sweeps conducted, the estimated range for symptomatic infection cases is [1,669, 1,750]. Adjusting this number to the comparison group with a higher population, the estimated range for symptomatic infection cases is [2,105, 2,208].

Our simulation results show that about 90% of the infection cases occurred during peak season. Considering this, the estimated range for year-round infection cases is [2338, 2,454]. The symptomatic

infection cases reported in Table S8 (0.496*4747=2,354) lies in the range of year-round infection cases. Moreover, the total number of positive RDTs given during sweeps reported in Linn et al. is 647. The estimated number of positive RDTs in the comparison group after adjustment is 933, which lies in the 95% confidence interval of our results.

In addition to validating our model with the number symptomatic infection cases and positive RDTs from the pilot study, we also compare with the Plasmodium falciparum parasite rate (PfPR). The yearly average $Pf\mathrm{PR}_{0-5}$ from our model is 9.3%, which lies in the published range.[31,32]

To check that model sensitivities are roughly in keeping with expectations, we performed some trade-offs on ProCCM sweep coverage and simulation year. We adjusted the coverage from 80% to 95% while adopting weekly sweep strategy (C) and alternative strategy (I); the results are shown in Table S9 and Table S10. Since the weekly sweep strategy (strategy C in Table S8) provides higher frequency interventions, the results (number of malaria infection cases) are more sensitive to coverage change. Decreasing coverage from 100% to 80% would result in 8.7% more infection cases per year. When adopting a strategy with lower intervention frequency (strategy I in Table S8), 6.3% more infection cases result per year.

We repeated the simulation from 2010 to 2013 when adopting the following strategies: i) no sweeps conducted, ii) weekly sweeps conducted during transmission season and iii) alternative strategy (I) conducted. We compared the total number of symptomatic malaria infection cases per year in Table S11. The results show consistency from year to year: i) the total number of symptomatic infection cases per year is around 3700 in sweep strategy A (i.e., comparison group); ii) conducting weekly sweeps could reduce 31% to 36% of year-round infection cases; iii) conducting alternative strategy (I) could reduce 27% to 31% of year-round infection cases, while reducing 33% of the implementation cost.

Table S8 Simulation results for all test cases in year 2013

Test Cases	Total Symptomatic Infection [incidence per person]	SD	Peak Weekly Symptomatic Prevalence [max infection cases per person]	SD	RDTs performed	SD
A. No Sweeps [0]	0.779	0.03	0.0536	0.002	0	0
B. 3 Sweeps, 100% coverage [3]	0.628	0.02	0.0416	0.002	185	8
C. Weekly Sweeps, 100% coverage of symptomatic individuals [21]	0.496	0.03	0.0200	0.001	945	56
D. Weekly Sweeps, 50% coverage [21]	0.602	0.02	0.0279	0.001	594	23
E. Bi-weekly Sweeps, 100% coverage [11]	0.569	0.03	0.0304	0.002	578	32
F. Twice a week, 100% coverage [41]	0.389	0.03	0.0154	0.001	1159	87
G. Weekly Sweeps for first 6 weeks, 100% coverage [7]	0.570	0.02	0.0361	0.002	235	21
G*. Weekly Sweeps for 6 weeks during mid-season, 100% coverage [7]	0.675	0.02	0.0334	0.001	479	26
H. Bi-Weekly Sweeps for first 12 weeks, 100% coverage [7]	0.576	0.03	0.0339	0.002	299	24
I. Weekly sweep from week 5 to week 11, biweekly sweep at week 1, 3, 12, 14, 16, 18,20.[14]	0.541	0.02	0.0286	0.002	670	34
J. Weekly sweep from week 5 to week 11, biweekly sweep at week 3, 12, 14, 16. [11]	0.584	0.03	0.0301	0.002	551	38

SD = standard deviation

Table S9 Sensitivity analysis for 21 weekly sweeps with different coverage (C)*

Coverage	Total Symptomatic Infection [incidence per person]	Peak Weekly Symptomatic Prevalence [max infection cases per person]	RDT
**1**	0.492	0.0200	933
0.95	0.498	0.0206	900
0.9	0.518	0.0216	889
0.85	0.521	0.0224	846
0.8	0.535	0.0233	823

Table S10 Sensitivity analysis for alternative sweeping strategy I with different coverage (I)*

Coverage	Total Symptomatic Infection [incidence per person]	Peak Weekly Symptomatic Prevalence [max infection cases per person]	RDTs performed
1	0.537	0.028	666
0.95	0.539	0.029	629
0.9	0.559	0.029	628
0.85	0.560	0.029	592
0.8	0.571	0.030	571

Table S11 Total Infections for Strategies A, C, and I in multiple years.

Year	Total Symptomatic Infection (A) [incidence per person]	Total Symptomatic Infection (C) [incidence per person]	Total Symptomatic Infection (I) [incidence per person]
2010	0.733	0.410	0.438
2011	0.809	0.531	0.568
2012	0.797	0.533	0.566
2013	0.788	0.493	0.535

For most of the sub-Sahara African countries, funding for malaria intervention is a major barrier. Thus, we aim at exploring a better option that is more cost-efficient while providing promising results in infection control. Assuming each sweep has the same coverage, with the same amount of homecare providers, the average number of malaria infection cases identified per sweep would be an essential indicator of the cost efficiency for ProCCM strategies. Obviously, to not implement ProCCM at all would not induce any cost.

ProCCM sweeps started on July 8th and ended on Nov 25th of 2013 in the pilot study. To find the optimal starting date of sweeps, we first proposed 13 sweep strategies, all of which are composed of 7 consecutive weekly strategies and each of them starting on a different week during peak season; results of this analysis are reported in Table S12. We only selected 7 out of 21 sweeps in this comparison set to reduce the cost of intervention. By exhaustive search, we identified the optimal strategy with the current

constraints: strategy G test case #1 which starts on the 27th week and lasts until the end of the 32nd week. With 26.4% reduction in infection cases, it is still not comparable to having 21 weekly sweeps, but two-thirds of the cost is reduced. We repeated the analysis on years 2010, 2011 and 2012. In all three cases, the optimal starting weeks for 7 consecutive weekly sweeps are week 27, corresponding to strategy G. Strategy G test cases #5 to #13 are shown to be ineffective compared to strategy B, where only three sweeps are adopted. Since the peak of malaria prevalence is reached at the mid-late peak season, starting intervention during mid-peak season is not effective when the malaria prevalence within the mosquito population is already very high. Early intervention is needed for effective malaria control. More details about yearly number of symptomatic and asymptomatic cases of all ages and max prevalence for this weekly sweeps strategy can be found in Figures S5-S7.

Table S12 Simulation results for 7 consecutive weekly sweeps with different starting dates (variations to Strategy G)

Weekly Sweeps (2013)							
**Test Cases**	**Sweep Duration**	**Total Symptomatic Infection [incidence per person]**	**SD**	**Peak Weekly Symptomatic Prevalence [max infection cases per person]**	**SD**	**RDTs performed**	**SD**
1	07/08-08/19 [7]	0.573	0.030	0.036	0.0014	238	27
2	07/15-08/26 [7]	0.598	0.025	0.036	0.0015	280	24
3	07/22-09/02 [7]	0.615	0.027	0.036	0.0018	316	23
4	07/29-09/09 [7]	0.626	0.024	0.035	0.0015	347	28
5	08/05-09/16 [7]	0.644	0.025	0.035	0.0015	390	29
6	08/12-09/23 [7]	0.665	0.023	0.034	0.0014	440	26
7	08/19-09/30 [7]	0.667	0.027	0.034	0.0013	470	30
8	08/26-10/07 [7]	0.690	0.025	0.033	0.0015	515	25
9	09/02-10/14 [7]	0.698	0.026	0.031	0.0017	547	28
10	09/09-10/21 [7]	0.711	0.024	0.033	0.0024	584	20
11	09/16-10/28 [7]	0.728	0.023	0.036	0.0024	615	20
12	09/23-11/04 [7]	0.740	0.025	0.040	0.0026	639	21
13	09/30-11/11 [7]	0.740	0.023	0.041	0.0019	647	16

SD = standard deviation

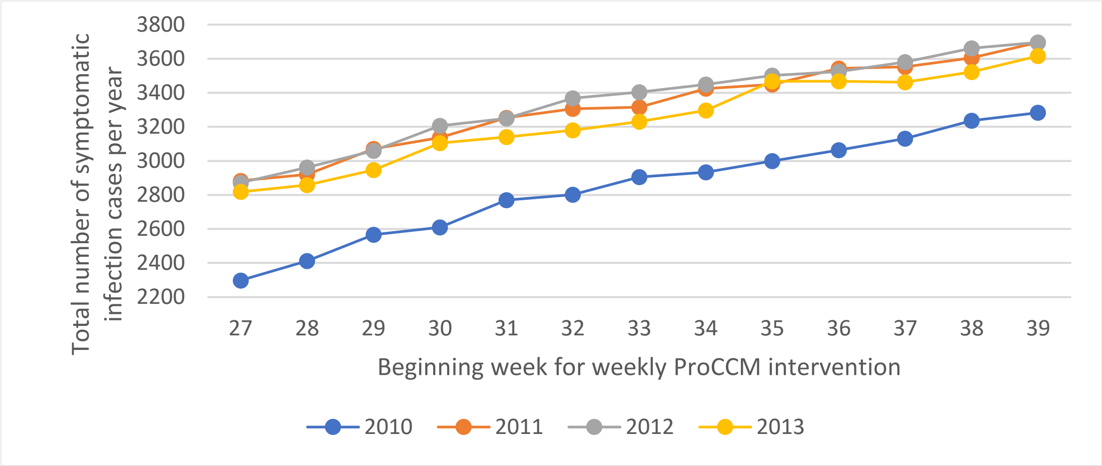

Figure S5 Sum of symptomatic infection cases per year for 7 consecutive weekly sweeps with different starting dates

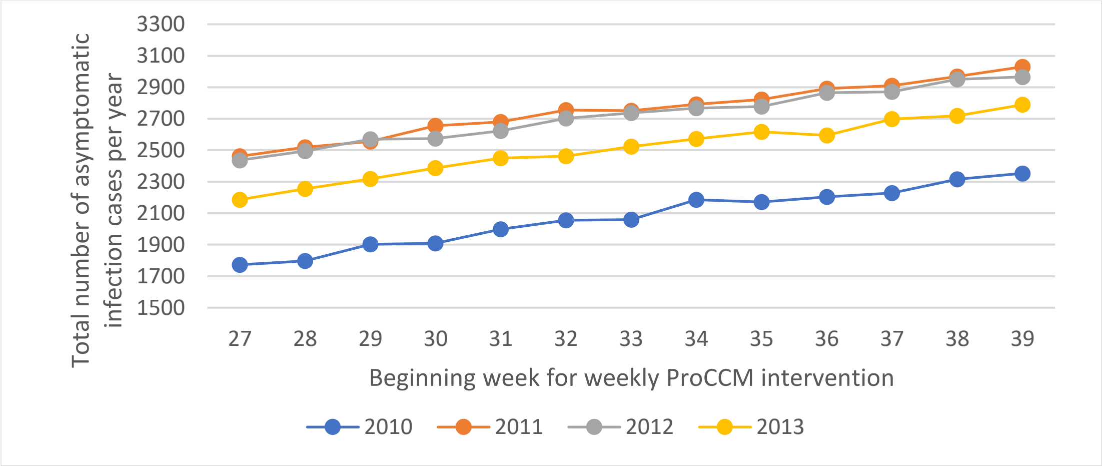

Figure S6 Sum of asymptomatic infection cases per year for 7 consecutive weekly sweeps with different starting dates

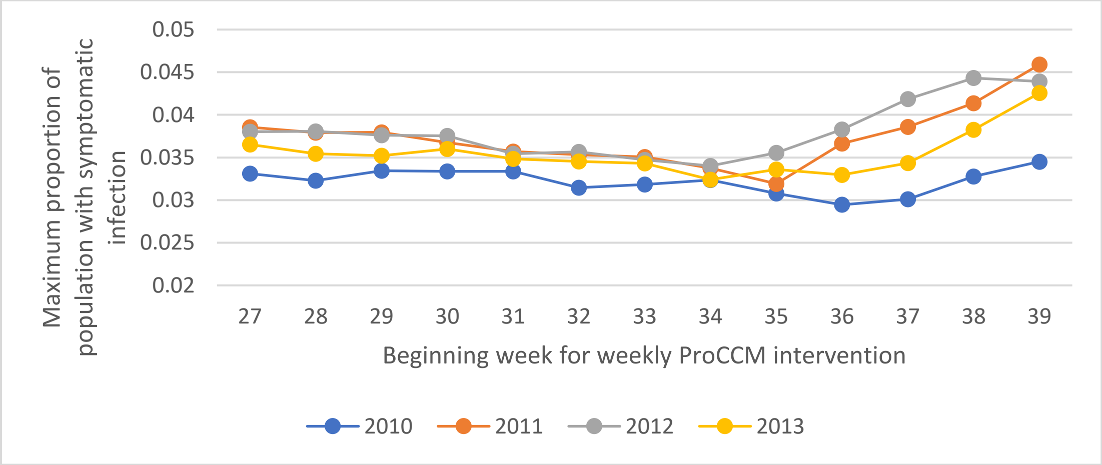

Figure S7 Maximum proportion of population with symptomatic infection weekly under different starting dates

Next, we examined the effect of having a longer intervention period by using biweekly sweeps. Strategy E conducts 11 biweekly sweeps converging with the same 21-week period starting on July 8th. The biweekly strategy could reduce the cost by half while still following the requirement of an early start and long duration. Alternatively, Strategy D conducts 21 weekly sweeps but each time only having 50% population coverage instead of 100%. Reduction in malaria infections in both cases are similar, with strategy D performing slightly better. Under Strategy D, there are infectious humans who were detected earlier than under the biweekly sweeps due to randomness. Theoretically having 50% coverage each sweep could also reduce the cost by half, yet the idea of randomly selecting 50% of the symptomatic humans to test is hard to implement. Covering half of the villages on odd weeks and the other half on even weeks in more likely to be carried out in practice. Without the randomness in the selection, the outcome is going to be different.

Within the low-cost setting, we examined the optimal starting date for 7 consecutive biweekly sweeps in years 2010, 2011, 2012 and 2013 (results for year 2013 are reported in Table S13). In all four cases, the optimal starting week for 7 consecutive biweekly sweeps is at the beginning of peak season on week 27, corresponding to strategy test case H. The weekly and biweekly strategies with only 7 sweeps perform comparably in terms of outcomes, but higher frequency, especially at the beginning of the transmission season, is preferred. More details about yearly number of symptomatic and asymptomatic cases of all ages and max prevalence for this biweekly sweeps strategy can be found in Figures S8 - S10.

Table S13 Simulation results for 7 consecutive biweekly sweeps with different starting dates (variations to Strategy H)

Biweekly Sweeps (2013)							
**Test Cases**	**Sweep Duration**	**Total Symptomatic Infection [incidence per person]**	**SD**	**Peak Weekly Symptomatic Prevalence [max infection cases per person]**	**SD**	**RDTs Performed**	**SD**
1	07/08-09/30[7]	0.576	0.026	0.0340	0.0015	295	22
2	07/15-10/07 [7]	0.596	0.026	0.0333	0.0016	337	26
3	07/22-10/14 [7]	0.625	0.028	0.0320	0.0015	384	29
4	07/29-10/21 [7]	0.641	0.030	0.0321	0.0017	429	28
5	08/05-10/28 [7]	0.659	0.029	0.0320	0.0010	467	24
6	08/12-11/04 [7]	0.684	0.027	0.0320	0.0014	511	26
7	08/19-11/11 [7]	0.688	0.022	0.0321	0.0013	534	20

SD = standard deviation

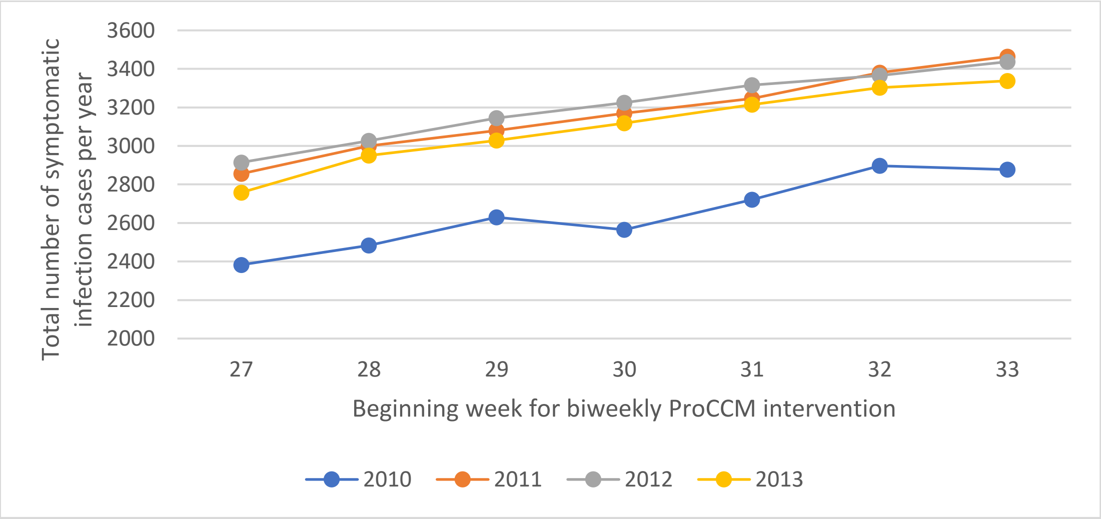

Figure S8 Sum of symptomatic infection cases per year for 7 consecutive bi-weekly sweeps with different starting dates'

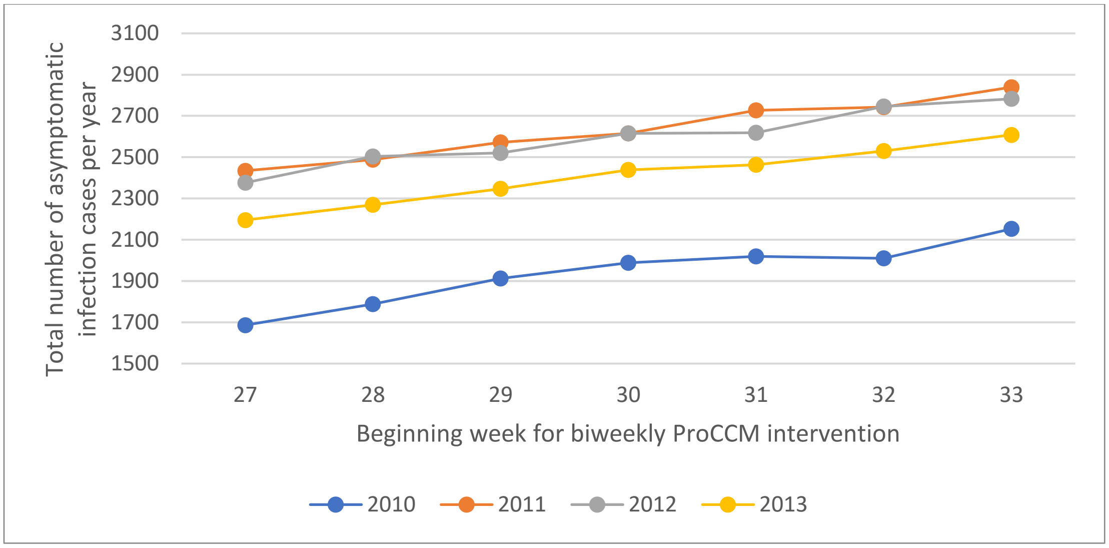

Figure S9 Sum of asymptomatic infection cases per year for 7 consecutive bi-weekly sweeps with different starting dates

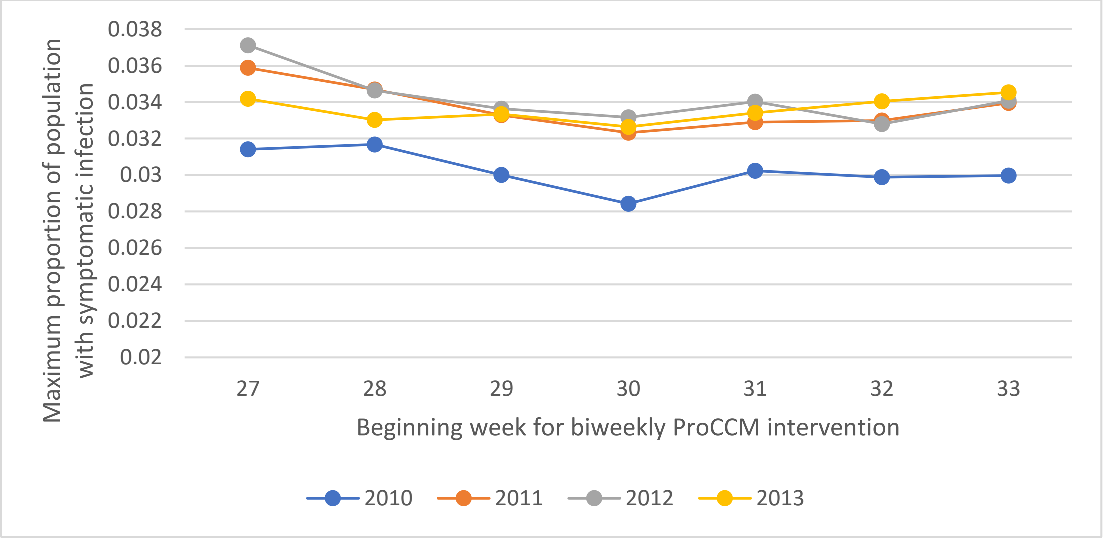

Figure S10 Maximum proportion of population with symptomatic infection weekly under different starting dates

We performed sensitivity analyses to determine the impact of variations to the treatment seeking rate parameters incorporated in the model; results in Figures S11-S12 correspond to the number of symptomatic cases yearly and cases detected by sweeps for Strategy C: weekly sweeps. In the simulation, the daily treatment seeking rate at mild condition and severe condition are assumed to be 0.25 and 0.60, respectively.

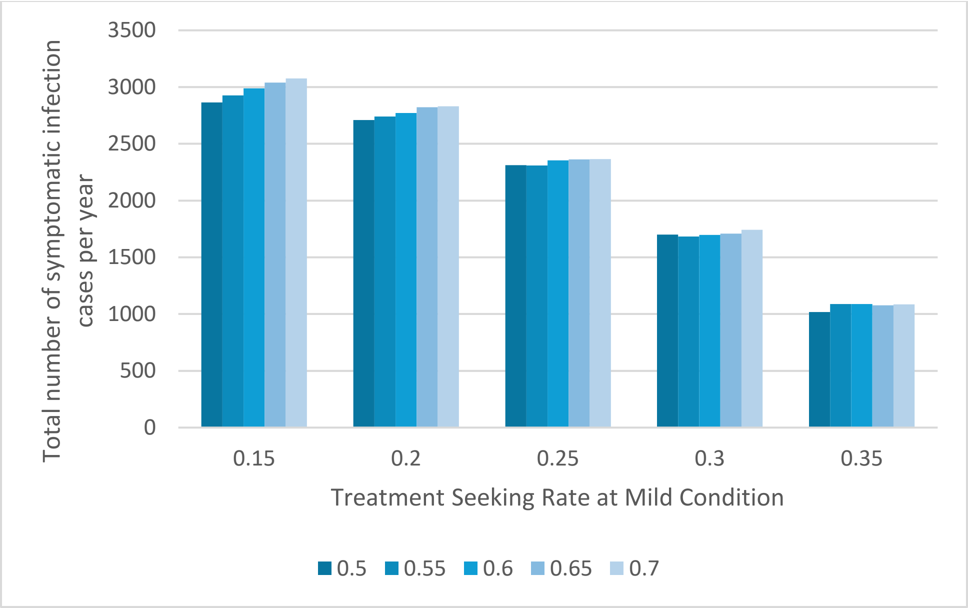

Figure S11 The total number of symptomatic infection cases per year for Strategy C: weekly sweeps, given variations in treatment seeking rates at mild condition and severe condition, respectively. The legend corresponds to treatment seeking rates at severe condition.

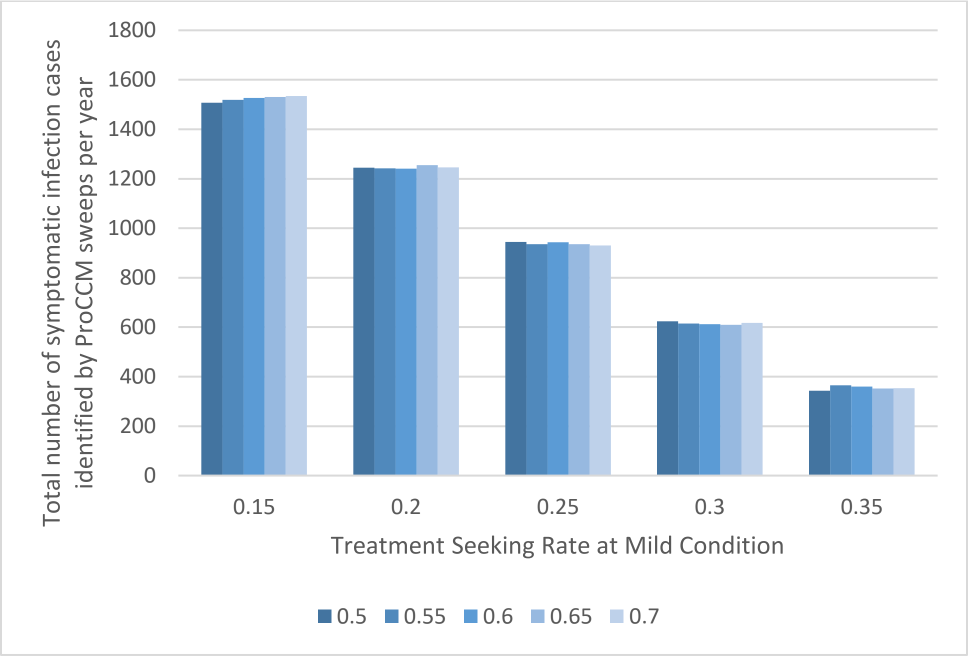

Figure S12 The total number of symptomatic infection cases identified by ProCCM sweeps per year for Strategy C: weekly sweeps, given variations in treatment seeking rates at mild condition and severe condition, respectively. The legend corresponds to treatment seeking rates at severe condition.

Note that the simulation stabilizes after approximately 40 simulation replications; for example, the rolling mean and standard deviation of symptomatic cases for Strategy C (i.e., weekly sweeps) are shown in Figures S13 and S14, respectively.

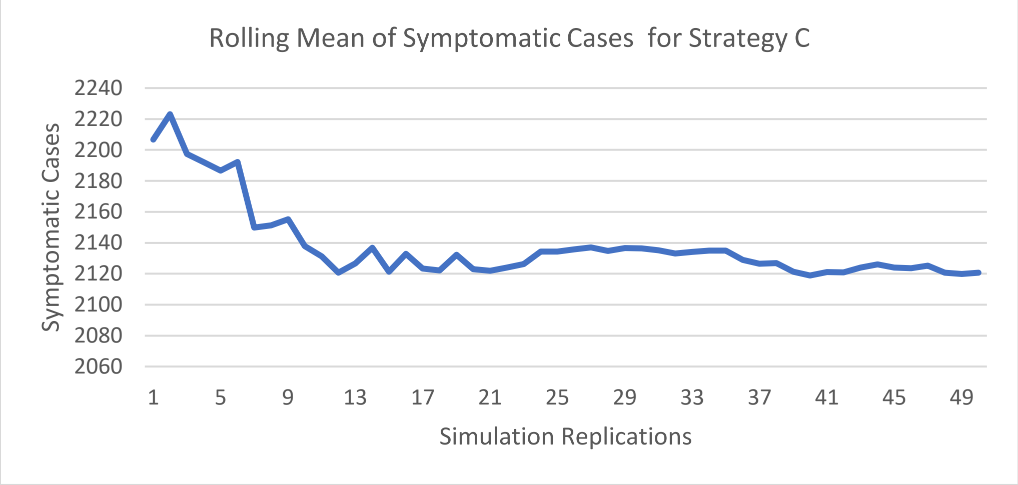

Figure S13 Rolling mean of symptomatic cases for Strategy C (i.e., weekly sweeps) for simulation iterations up to 50.

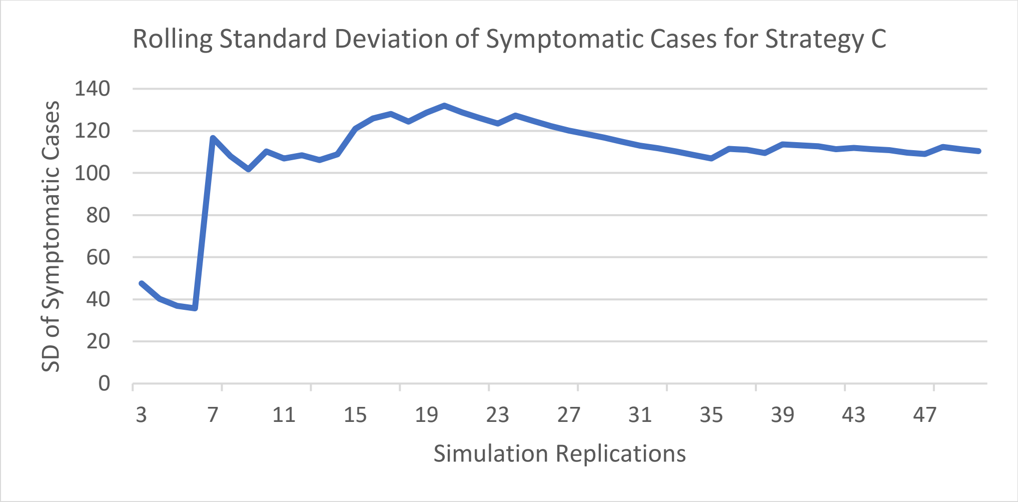

Figure S14 Rolling standard deviation of symptomatic cases for Strategy C (i.e., weekly sweeps) for simulation iterations up to 50.

Section 4. Limitations

There are parameters in our human infection transition model that are estimated within certain published ranges. We adopted parameters that fit our model the best and provided the closest result to existing data in Linn et al[4]. To test the robustness of our conclusions, we chose varying values of environment carrying capacity and reran the simulation. As shown in Table S14, despite different environment capacities and the resulting reductions of symptomatic cases per year, strategy I is at least as effective as strategy C while reducing the number of sweeps.

This robustness test can be regarded as a thumbnail of the large-scale experiment involving other parameters. Different parameters lead to different transmission intensity, which can be measured by entomological inoculation rate (EIR), as shown in Table S15. Within a certain range of transmission intensity, strategy I highlights its advantage in balancing resources and effectiveness.

Table S14 Mean Reduction of symptomatic cases (from 0.0 to 1.0) per year (compared to A, no sweeps) for various strategies with different Larvae and pupae environment carrying capacity

	Environment Capacity (Larvae and Pupae) (*10000)				
**Test Cases**	**25**	**30**	**35**	**40**	**45**
B	0.355	0.246	0.191	0.154	0.109
C	0.608	0.473	0.368	0.275	0.215
D	0.431	0.312	0.240	0.181	0.131
E	0.494	0.364	0.274	0.209	0.160
F	0.742	0.609	0.508	0.402	0.311
G	0.493	0.361	0.273	0.209	0.157
G*	0.273	0.174	0.138	0.097	0.080
H	0.464	0.343	0.260	0.200	0.158
I	0.533	0.412	0.318	0.243	0.188
J	0.441	0.333	0.251	0.189	0.145

Table S15 Mean Daily EIR during the high transmission season for various strategies with different Larvae and pupae environment carrying capacity

	Environment Capacity (Larvae and Pupae) (*10000)				
**Test Cases**	**25**	**30**	**35**	**40**	**45**
B	0.025	0.043	0.063	0.082	0.101
C	0.013	0.026	0.039	0.053	0.069
D	0.007	0.015	0.024	0.036	0.047
E	0.011	0.022	0.034	0.047	0.061
F	0.009	0.019	0.031	0.044	0.057
G	0.004	0.010	0.016	0.025	0.035
G*	0.010	0.020	0.033	0.047	0.061
H	0.015	0.028	0.042	0.056	0.070
I	0.010	0.020	0.033	0.046	0.059
J	0.008	0.017	0.028	0.040	0.052

References

1. Deressa W. Treatment-seeking behaviour for febrile illness in an area of seasonal malaria transmission in rural Ethiopia. *Malaria Journal.* 2007;6(1):1-7.
2. Lindblade KA, Steinhardt L, Samuels A, Kachur SP, Slutsker L. The silent threat: asymptomatic parasitemia and malaria transmission. *Expert review of anti-infective therapy.* 2013;11(6):623-639.
3. NCDC Climate Data Online. 2018. https://www7.ncdc.noaa.gov/CDO/cdoselect.cmd.
4. Linn AM, Ndiaye Y, Hennessee I, et al. Reduction in symptomatic malaria prevalence through proactive community treatment in rural Senegal. *Tropical Medicine & International Health.* 2015;20(11):1438-1446.
5. Agence Nationale de la Statistique et de la Démographie - ANSD/Sénégal, ICF International. *Sénégal : Enquête Démographique et de Santé Continue (EDS-Continue 2017).* Rockville, Maryland, USA: ANSD/Sénégal and ICF International;2018.
6. Cailly P, Tran A, Balenghien T, L'Ambert G, Toty C, Ezanno P. A climate-driven abundance model to assess mosquito control strategies. *Ecological Modelling.* 2012;227:7-17.
7. Tran A, L'ambert G, Lacour G, et al. A rainfall-and temperature-driven abundance model for Aedes albopictus populations. *International journal of environmental research and public health.* 2013;10(5):1698-1719.
8. Winskill P, Slater HC, Griffin JT, Ghani AC, Walker PG. The US president's malaria initiative, Plasmodium falciparum transmission and mortality: a modelling study. *PLoS Medicine.* 2017;14(11):e1002448.
9. Rodriguez-Barraquer I, Arinaitwe E, Jagannathan P, et al. Quantification of anti-parasite and anti-disease immunity to malaria as a function of age and exposure. *Elife.* 2018;7:e35832.
10. Ashley EA, Pyae Phyo A, Woodrow CJ. Malaria. *The Lancet.* 2018;391(10130):1608-1621.
11. WHO. Severe Malaria. *Tropical Medicine & International Health.* 2014;19(s1):7-131.
12. Trampuz A, Jereb M, Muzlovic I, Prabhu RM. Clinical review: Severe malaria. *Critical care.* 2003;7(4):1-9.
13. Bradley J, Stone W, Da DF, et al. Predicting the likelihood and intensity of mosquito infection from sex specific Plasmodium falciparum gametocyte density. *Elife.* 2018;7.
14. Beshir KB, Sutherland CJ, Sawa P, et al. Residual Plasmodium falciparum parasitemia in Kenyan children after artemisinin-combination therapy is associated with increased transmission to mosquitoes and parasite recurrence. *The Journal of infectious diseases.* 2013;208(12):2017-2024.
15. Silva-Nunes Md, Ferreira MU. Clinical spectrum of uncomplicated malaria in semi-immune Amazonians: beyond the" symptomatic" vs" asymptomatic" dichotomy. *Memórias do Instituto Oswaldo Cruz.* 2007;102:341-348.
16. Langhorne J, Ndungu FM, Sponaas A-M, Marsh K. Immunity to malaria: more questions than answers. *Nature immunology.* 2008;9(7):725-732.
17. Bartoloni A, Zammarchi L. Clinical aspects of uncomplicated and severe malaria. *Mediterranean journal of hematology and infectious diseases.* 2012;4(1).
18. Mandal S, Sarkar RR, Sinha S. Mathematical models of malaria-a review. *Malaria journal.* 2011;10(1):1-19.
19. Sumba PO, Wong SL, Kanzaria HK, Johnson KA, John CC. Malaria treatment-seeking behaviour and recovery from malaria in a highland area of Kenya. *Malaria Journal.* 2008;7(1):1-8.
20. Thwing J, Eisele TP, Steketee RW. Protective efficacy of malaria case management and intermittent preventive treatment for preventing malaria mortality in children: a systematic review for the Lives Saved Tool. *BMC Public Health.* 2011;11.

21. Snow RW, Craig M, Deichmann U, Marsh K. Estimating mortality, morbidity and disability due to malaria among Africa's non-pregnant population. *Bulletin of the World Health Organization.* 1999;77(8):624.
22. Doolan DL, Dobaño C, Baird JK. Acquired immunity to malaria. *Clinical microbiology reviews.* 2009;22(1):13-36.
23. Cailly P, Balenghien T, Ezanno P, Fontenille D, Toty C, Tran A. Role of the repartition of wetland breeding sites on the spatial distribution of Anopheles and Culex, human disease vectors in Southern France. *Parasites & Vectors.* 2011;4(1):1-8.
24. Bomblies A. Agent-based modeling of malaria vectors: the importance of spatial simulation. *Parasites & vectors.* 2014;7(1):1-10.
25. Isaïa J, Rivero A, Glaizot O, Christe P, Pigeault R. Last-come, best served? Mosquito biting order and Plasmodium transmission. *Proceedings of the Royal Society B.* 2020;287(1939):20202615.
26. Sequeira J, Louçã J, Mendes AM, Lind PG. A model for assessing the quantitative effects of heterogeneous affinity in malaria transmission along with ivermectin mass administration. *Applied Sciences.* 2020;10(23):8696.
27. Ngwa GA, Shu WS. A mathematical model for endemic malaria with variable human and mosquito populations. *Mathematical and computer modelling.* 2000;32(7-8):747-763.
28. Filipe JAN, Riley EM, Drakeley CJ, Sutherland CJ, Ghani AC. Determination of the Processes Driving the Acquisition of Immunity to Malaria Using a Mathematical Transmission Model. *PLOS Computational Biology.* 2007;3(12):e255.
29. Achan J, Tibenderana JK, Kyabayinze D, et al. Effectiveness of quinine versus artemether-lumefantrine for treating uncomplicated falciparum malaria in Ugandan children: randomised trial. *Bmj.* 2009;339.
30. Bousema T, Okell L, Felger I, Drakeley C. Asymptomatic malaria infections: detectability, transmissibility and public health relevance. *Nature Reviews Microbiology.* 2014;12(12):833-840.
31. Noor AM, Kinyoki DK, Mundia CW, et al. The changing risk of Plasmodium falciparum malaria infection in Africa: 2000–10: a spatial and temporal analysis of transmission intensity. *The Lancet.* 2014;383(9930):1739-1747.
32. Agence Nationale de la Statistique et de la Démographie , International I. Senegal Demographic and Health and Multiple Indicator Cluster Survey (EDS-MICS) 2010–2011. ANSD and ICF International Rockville; 2012.
